# Obesity alters the estrogen-induced luteinizing hormone surge in ovariectomized ewes^†^

**DOI:** 10.1093/biolre/ioag135

**Published:** 2026-07-01

**Authors:** Trent Bronnenberg, Kelly S Kirkley, Christianne Magee, Dilyara A Murtazina, Whit C Stewart, Terry M Nett, Amy M Navratil, Jeremy D Cantlon, Brian D Cherrington, Colin M Clay

**Affiliations:** Department of Zoology and Physiology, University of Wyoming, Laramie, WY 82071, USA; Department of Biomedical Sciences, Colorado State University, Fort Collins, CO 80523, USA; Department of Biomedical Sciences, Colorado State University, Fort Collins, CO 80523, USA; Department of Biomedical Sciences, Colorado State University, Fort Collins, CO 80523, USA; Department of Animal Science, University of Wyoming, Laramie, WY 82071, USA; Department of Biomedical Sciences, Colorado State University, Fort Collins, CO 80523, USA; Department of Zoology and Physiology, University of Wyoming, Laramie, WY 82071, USA; Department of Biomedical Sciences, Colorado State University, Fort Collins, CO 80523, USA; Department of Zoology and Physiology, University of Wyoming, Laramie, WY 82071, USA; Department of Biomedical Sciences, Colorado State University, Fort Collins, CO 80523, USA

**Keywords:** pituitary, gonadotrope, estradiol positive feedback, sheep, hyperinsulinemia, gonadotropin-releasing hormone receptor, pituitary sensitivity, reprometabolic syndrome, metabolic regulation of reproduction, neuroendocrine regulation

## Abstract

Obesity is a leading health issue with a negative effect on fertility, but the complexity of the relationship between obesity and fertility requires further study. Santoro and colleagues have characterized the metabolic environment of obesity and the development of reprometabolic syndrome. Due to the reproductive similarities between ewes and women, we use ovariectomized ewes as a translational model of reprometabolic syndrome and tested whether obesity alters the estradiol-17β (E2)-induced luteinizing hormone (LH) surge. Each ovariectomized ewe serves as her own biological control during the three phases of the study: normal weight (phase 1), obese (phase 2), and a return to normal weight (phase 3). Ewes were weighed weekly, and 12th rib fat thickness was measured as a proxy for body condition score. During each of the three phases of the study, following an intramuscular injection of 25 μg of E2, serial blood samples were collected, and serum LH concentrations were measured by radioimmunoassay. The peak concentration and timing of the LH surge are significantly lower and delayed in obese ewes in phase 2 as compared to phases 1 and 3. Our results suggest that the mechanisms that underlie the pathological effects of obesity and reprometabolic syndrome on the E2-induced LH surge are similar in ewes and women. Therefore, ewes can serve as a model for parsing the relative roles of diminished hypothalamic and pituitary sensitivity to E2, resulting in a reduced and delayed LH surge associated with obesity and reprometabolic syndrome in women.

## Teaser

Diet-induced obesity with hyperinsulinemia delays and attenuates the estradiol-induced LH surge in ewes, recapitulating key endocrine reproductive features of obesity in women.

## Graphical Abstract

**Graphical Abstract:**
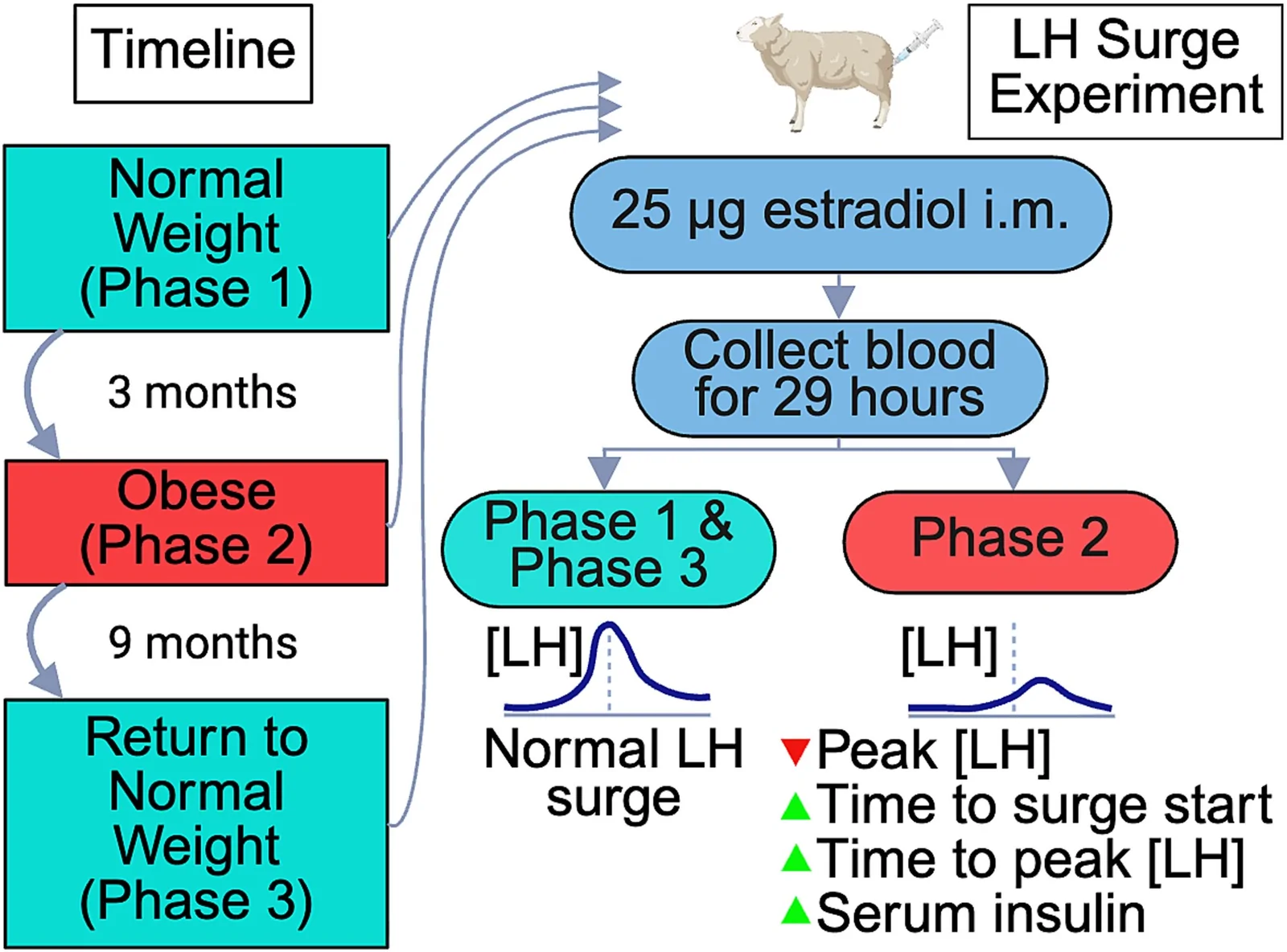

## Introduction

Obesity has emerged as one of the leading health issues not only in the United States, where it is estimated that >30% of the population is clinically obese [1] but also globally [2]. Obesity is associated with a wide array of poor health outcomes, including diminished reproductive fitness. In women, obesity causes a relatively hypogonadotropic hypogonadal phenotype, characterized by reduced secretion of luteinizing hormone (LH) and follicle-stimulating hormone (FSH) [3–5]. Additionally, decreased gonadotropin secretion associated with obesity is related to reduced pituitary sensitivity to gonadotropin-releasing hormone (GnRH) [6] and decreased LH pulse amplitude [3, 7, 8]. Santoro and colleagues have named this phenotype reprometabolic syndrome. This syndrome includes infertility characterized by menstrual irregularities and a prolonged follicular phase, suggestive of ovulatory deficiencies [5, 8, 9] associated with hyperinsulinemia and elevated free fatty acids [10]. The reduced LH pulse amplitude and blunted response to GnRH in women with obesity implicate a functional impairment of the hypothalamic–pituitary–gonadal (HPG) axis that is manifested at the level of the pituitary gland. A similar phenotype has been noted for nonhuman primates fed a high-fat diet [11]. Despite the profound impacts of obesity on reproductive function, the specific hypothalamic and pituitary defects that underlie this phenotype remain poorly defined. Due to the relative ease of genetic manipulation, rodents remain a key model for unraveling the complexities of the HPG; however, there are clear species differences in the organization of the neuronal pathways that underlie both pulsatile LH secretion and the preovulatory LH surge, limiting their translational relevance [12–15]. For example, Neurokinin-B contributes to generation of the estradiol-17β (E2)-induced LH surge in sheep and, perhaps, humans but not rodents [16–20]. Similarly, the key role of E2 to enhance pituitary sensitivity to GnRH in advance of the LH surge is well established in nonhuman primates [21] and sheep [22–24]. Therefore, we sought to determine whether sheep recapitulate key physiological features of reprometabolic syndrome in women, thereby establishing them as translational model.

## Materials and methods

### Ewe management and diet

**Figure 1:**
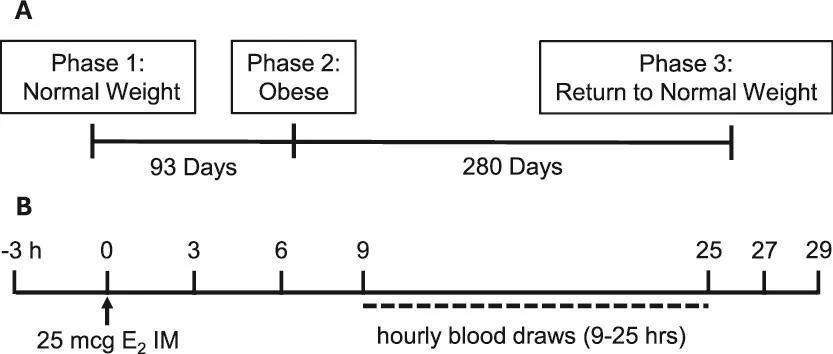
Experimental design and blood sampling schedule. (A) The experimental paradigm included three phases. Normal-weight ewes were fed a standard maintenance diet from OVX to phase 1 and then fed a high plane of nutrition to induce obesity for phase 2, followed by a return to a standard maintenance diet and a return to normal weight for phase 3. (B) An LH surge was induced by single IM dose of E2 (time 0), and blood samples were collected at depicted times.

All procedures and experiments were approved by the Colorado State University (CSU) and University of Wyoming (UW) Institutional Animal Care and Use Committees (CSU Protocol # 1233, UW Protocol # 20190618BC00377-01). All work complied with National Institutes of Health (NIH) guidelines. In the summer of 2019, 8 mature, western white-faced sheep (ewes) of a normal body weight (BW) were acquired and allowed a 2-week acclimation period prior to ovariectomy (OVX) at CSU. Following ovariectomy, ewes were allowed a minimum 6-month recovery period prior to transport to UW and initiation of the experiment. At UW, ewes were housed in a single drylot pen (6.4 × 36 m) with ad libitum access to water. Ewes were offered an alfalfa pellet diet (18% crude protein, 2.5 Mcal/kg dry matter basis) at approximately 1.75% of BW to meet maintenance nutrient requirements [25] for 7 days prior to blood sampling during phase 1 (normal weight). To achieve obesity (phase 2), ewes were offered the same alfalfa pellet, fed at 4.5% of their body weight for approximately 67 days prior to the phase 2 bleed. To return to normal weight (phase 3), ewes were again fed 1.75% of their body weight to achieve the desired weight loss intended by the study design. Phases 1 and 2 were conducted at UW prior to March 2020, while phase 3 was delayed and conducted at CSU once pandemic restrictions allowed for resuming research activities. The timing of phases 1–3 is shown in Figure 1A. Ewes were weighed weekly using a chute scale at UW and a portable livestock scale at CSU. Subcutaneous fat thickness was measured between the 12th and 13th rib using real-time B-mode ultrasonography (Aloka SSD-500V; Corometrics Medical Systems, Wallingford, CT) equipped with a 3.5-MHz, 12.5-cm linear-array transducer. Measurements were obtained by the same National Sheep Improvement Program-certified ultrasound technician in October 2019, January 2020, and August 2020, corresponding to body weight measurements during phases 1–3 of the study. Prior to image acquisition, the scanning site was shorn, and vegetable oil was applied to the skin as an acoustic coupling medium.

### E2 injection and hormone assays

Indwelling catheters (BD Angiocath; 16-gauge, 3.25 in; Becton Dickinson, Franklin Lakes, NJ) with stylets were placed in the external jugular vein under light sedation (ketamine, 1.5–3 mg/kg BW; diazepam, 0.25–0.8 mg/kg BW; IV) at least 8 h prior to E2 injection (time 0 h). Blood samples (8 ml sample with saline replacement) began with a sample taken 3 h prior to E2 injection. At 0 h, blood samples were taken and all ewes were given an intramuscular injection of 25 μg of E2 [26]. Thereafter, ewes were bled at 3 and 6 h, hourly from 9 to 25 h, and then at 27 and 29 h following E2 injection (Figure 1B). Blood was allowed to clot for 1 h at room temperature, and then serum was separated by centrifugation and stored at −20°C for subsequent quantification of hormones. Serum cytokines in blood samples collected at −3 h were assessed using a GS20 Cytokine Antibody Array (RayBiotech, Peachtree Corners, GA; #GSO-CAA-20-1) according to the manufacturer’s instructions. Insulin and cortisol were quantified by the Cornell University Animal Health and Diagnostic Center using radioimmunoassay methods for insulin and the Immulite 2000 Immunoassay system for cortisol. LH concentration was quantified by radioimmunoassay (RIA) at CSU as previously described [27]. The reference standard for LH was NIH-oLH-S13 (NHPP Lot# AFP5551B). Triplicate standard curves were included in each assay, and samples were analyzed in duplicate at 200 μl of sample per tube for LH. Intra-assay coefficients of variation for LH were 5.8%, while inter-assay coefficients of variation for LH were 14.7%. The minimum detectable amount of LH averaged 0.5 ng.

The effect of E2 treatment on the expected preovulatory-like surge of LH was evaluated using five parameters related to the release of LH: (1) LH concentration; (2) amplitude, defined as the highest concentration of LH; (3) area under the curve during the LH surge; (4) length of preovulatory-like LH surge; and (5) interval from the priming treatment to the beginning of the massive release of LH. The start of the LH surge was defined by an LH level that was greater than or equal to 2 standard deviations from baseline, followed by 2 consecutive increasing concentrations of LH. Baseline was calculated as the mean LH concentration of the −3 h and 0-h samples. The end of the surge was defined as the final sample with an LH concentration above baseline or the final sample if the criterion was not met.

### Data analysis

The experimental analysis compares the properties of the LH surge in the three consecutive phases associated with initial normal weight (phase 1), obesity (phase 2), and the return to normal weight (phase 3). The data from this repeated-measures design were analyzed using a linear mixed-effects model in R [28], using the lme4 package [29]. One model was fit for each LH surge. Each model animal state (phases 1–3) was included as a fixed effect, with random intercept for each ewe to account for repeated measures. Pairwise comparisons of the LH surge concentrations (ng/ml), peak time, start time, end time of the LH surge (hours), and area under the curve levels across groups were done using the emmeans package [30]. Emmeans (estimated marginal means, aka least-squares means) implemented t-tests of the difference using degrees of freedom calculated using the Kenward-Roger method and a Tukey adjustment for multiple comparisons. Area under the curve was calculated with trapz function from library pracma in R [31]. One ewe (#4) did not have a surge in phase 2 based on criteria (baseline +2 standard deviations from the baseline) and was excluded from the analysis in phase 2. One ewe (#1) was euthanized prior to the phase 3 blood collection for reasons unrelated to the experiment.

Body weight and back fat measurements were analyzed using similar linear mixed-effects models, with phase included as a fixed effect and ewe as a random effect. Post hoc comparisons were performed as described above. To satisfy the model assumption of normality, back fat measurements were log-transformed prior to analysis. Values are presented as back-transformed to the original scale.

## Results

### Development of a diet-induced obesity paradigm in ewes

Eight mature western white-faced OVX ewes were given an LH surge-inducing dose of E2 (25 μg IM). Ewes were bled 3 h prior to injection, just prior to E2 injection (0-h), at 3 and 6 h, hourly from 9 to 25 h, and then at 27 and 29 h. Serum LH concentrations were measured by RIA for phase 1 (normal body weight), phase 2 (obese), and phase 3 (return to a lean body weight) of the study, with each ewe serving as her own biological control (Figure 1A). The objective of this study was to test whether ewes can serve as a model to investigate reprometabolic syndrome.

### Body weight, 12th rib fat thickness, and serum insulin are elevated in obese ewes

**Figure 2:**
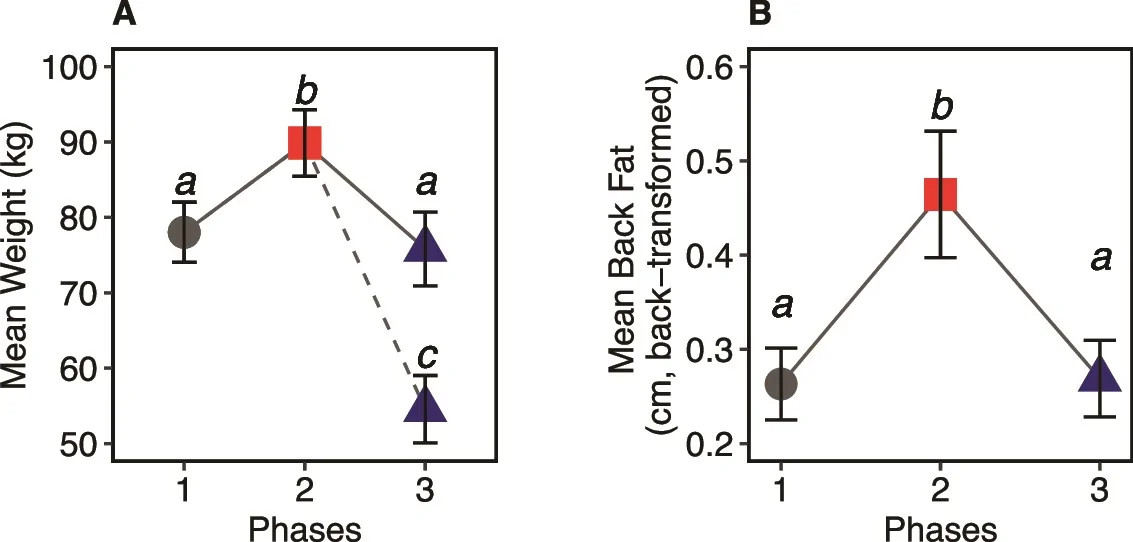
Body weight and back fat across experimental phases. (A) Body weight was measured weekly for phase 1 (normal weight, October 2019), phase 2 (obese, January 2020), and phase 3 (return to normal weight, October 2020). (B) Prior to sample collection in each phase, back fat was measured. The phase 3 back fat (2B) measures from August 2020 are matched to the solid line phase 3 weight (2A). The dashed line weight for phase 3 (2A) represents the actual weights at the time of the phase 3 E2-induced LH surge (October 2020). Data were analyzed using a linear mixed-effects model, as described in Materials and Methods. Values are back-transformed to the original scale. Different letters indicate significant differences between groups (*P* < 0.01).

**Figure 3:**
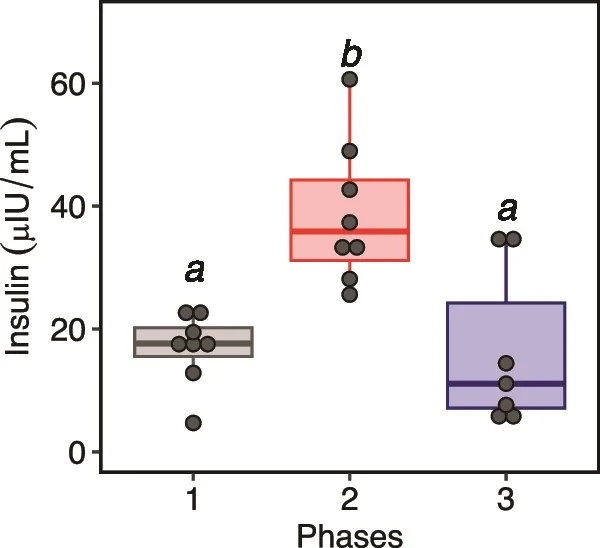
Obesity correlates with elevated serum insulin concentration. Blood insulin concentration was quantified from serum samples during each phase of the study. The boxes represent the interquartile range, with the line inside the box representing the median. Whiskers extending from the box represent the minimum and maximum values within 1.5× the IQR from first and third quartiles. The dots inside the boxes indicate individual ewes. Data was analyzed using linear mixed models as described in Materials and Methods. Significant differences between groups are indicated by different letters (*P* < 0.05).

Immediately prior to the phase 1 E2-induced LH surge, mean ewe BW was 78.0 ± 4.0 kg (Figure 2A), and subcutaneous fat thickness measured at the 12th rib by ultrasonography averaged 0.275 ± 0.031 cm (Figure 2B). At the end of phase 2, the obesity inducing diet caused an average gain of 11.9 ± 2.7 kg, resulting in a final average weight of 89.9 ± 4.4 kg, and added an average of 0.208 ± 0.033 cm of 12th rib fat thickness for a combined measurement of 0.482 ± 0.049 cm (Figure 2B). For phase 3, the ewes lost an average of 36.3 ± 0.89 kg for a mean weight of 54.6 ± 4.5 kg (Figure 2A, dotted line) and an average of 0.16 ± 0.04 cm of 12th rib fat thickness with a final average value of 0.31 ± 0.06 cm (Figure 2B). To confirm the metabolic effects of weight gain and subsequent loss, serum insulin was measured in the phases 1–3 0-hour serum sample. Similar to findings in women with reprometabolic syndrome, insulin levels were significantly elevated in phase 2 obese ewes (38.7 ± 4.1 μIU/ml) as compared to their phase 1 (16.8 ± 2.1 μIU/ml) and phase 3 (16.3 ± 4.9 μIU/mL) samples (Figure 3). Collectively, these findings suggest that diet-induced obesity results in a significant increase in ewe body weight, 12th rib fat thickness, and serum insulin, and support the validity of this model to study obesity-induced changes in the E2-mediated LH surge.

### Ewes display individual variation in LH surge dynamics

**Figure 4:**
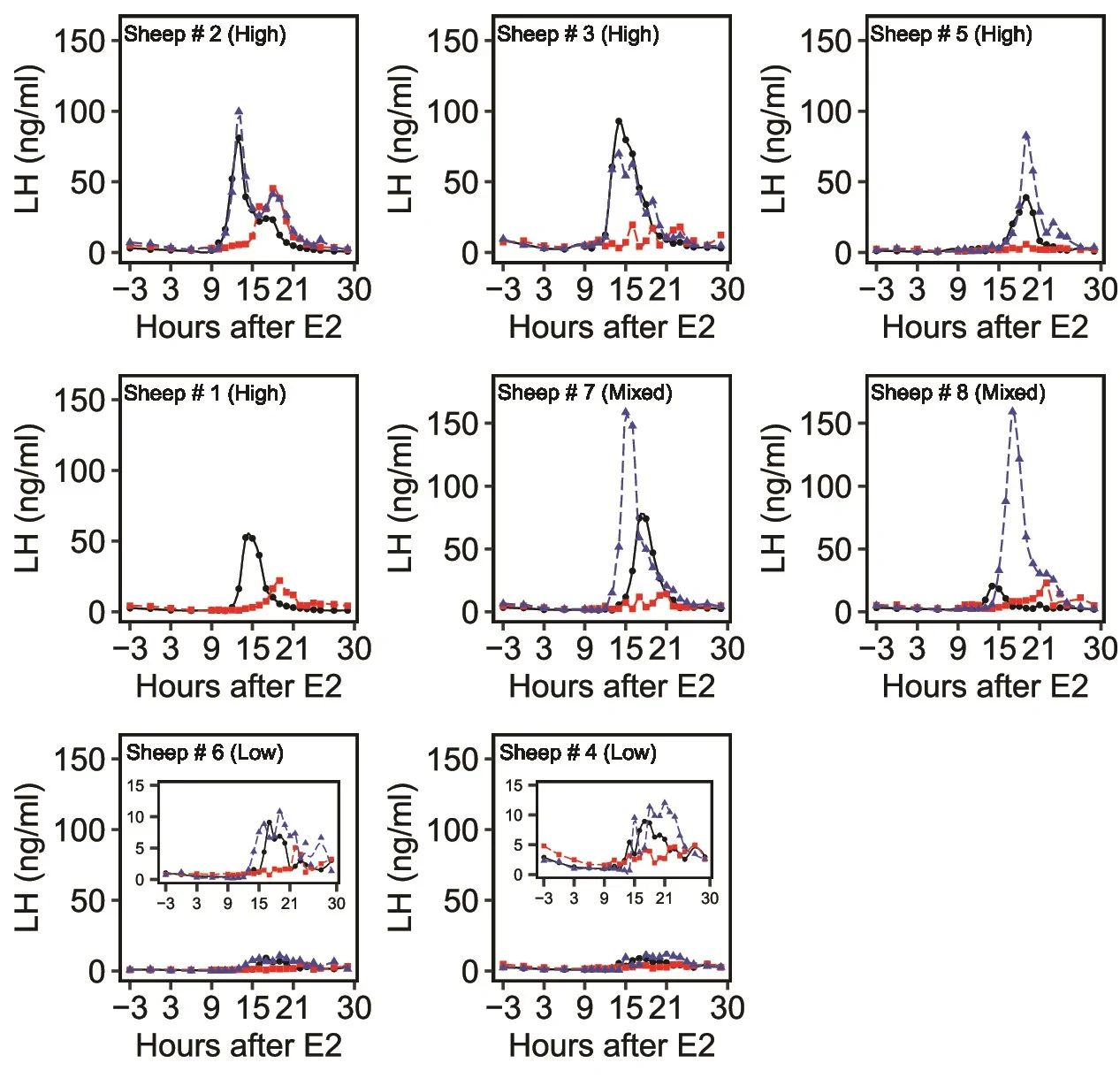
Ewes display individual variation in LH surge dynamics. Blood samples were taken over 29 h following IM injection of 25 μg E2 at 0 hours, and LH concentration quantified by RIA for each ewe during phase 1 (black line), phase 2 (red line), and phase 3 (blue line). High responders showed a large response to E2 during the phase 1 and phase 3, while mixed responders showed varied LH dynamics in response to E2 across phases. Low/No responders showed minimal LH response to E2 during all phases.

Serum LH concentration was quantified in each ewe during phases 1–3 by RIA. Based on qualitative evaluation of surge dynamics, including start time, peak LH concentration, and time of LH peak, ewes are divided into three groups: High, Mixed, and Low/No responders (Figure 4). Following E2 administration during phase 1 (black line), the four high responders (ewes 1, 2, 3, and 5) exhibited robust LH surges with a mean peak concentration of 66.4 ± 12.5 ng/ml at 15 ± 1.35 h post-administration. In phase 2 (red line), high responders had a visibly delayed surge start time (18 ± 0.7 h), lower peak LH concentration (23.2 ± 8.2 ng/ml), and time of LH peak as compared to phase 1. At phase 3 (blue line), high responders displayed E2-induced LH surges similar to those at phase 1 (LH surge start time 15.3 ± 1.9 h, LH peak concentration 84.1 ± 8.6 ng/ml, Figure 4). Ewe 1 was euthanized prior to phase 3 experiment. Mixed responders (ewes 7 and 8) had higher peak LH concentrations at phase 3 than during phase 1 or 2 (Figure 4). Finally, the low/no responders (ewe numbers 4 and 6) had low LH peak concentration, with ewe # 4 that did not have LH surge at phase 2 (Figure 4). Not only are LH surge dynamics altered in obese ewes, but these data are also consistent with the reduced urinary LH observed in obese women.

### Obesity alters LH surge dynamics, but these parameters are restored to normal with weight loss

**Figure 5:**
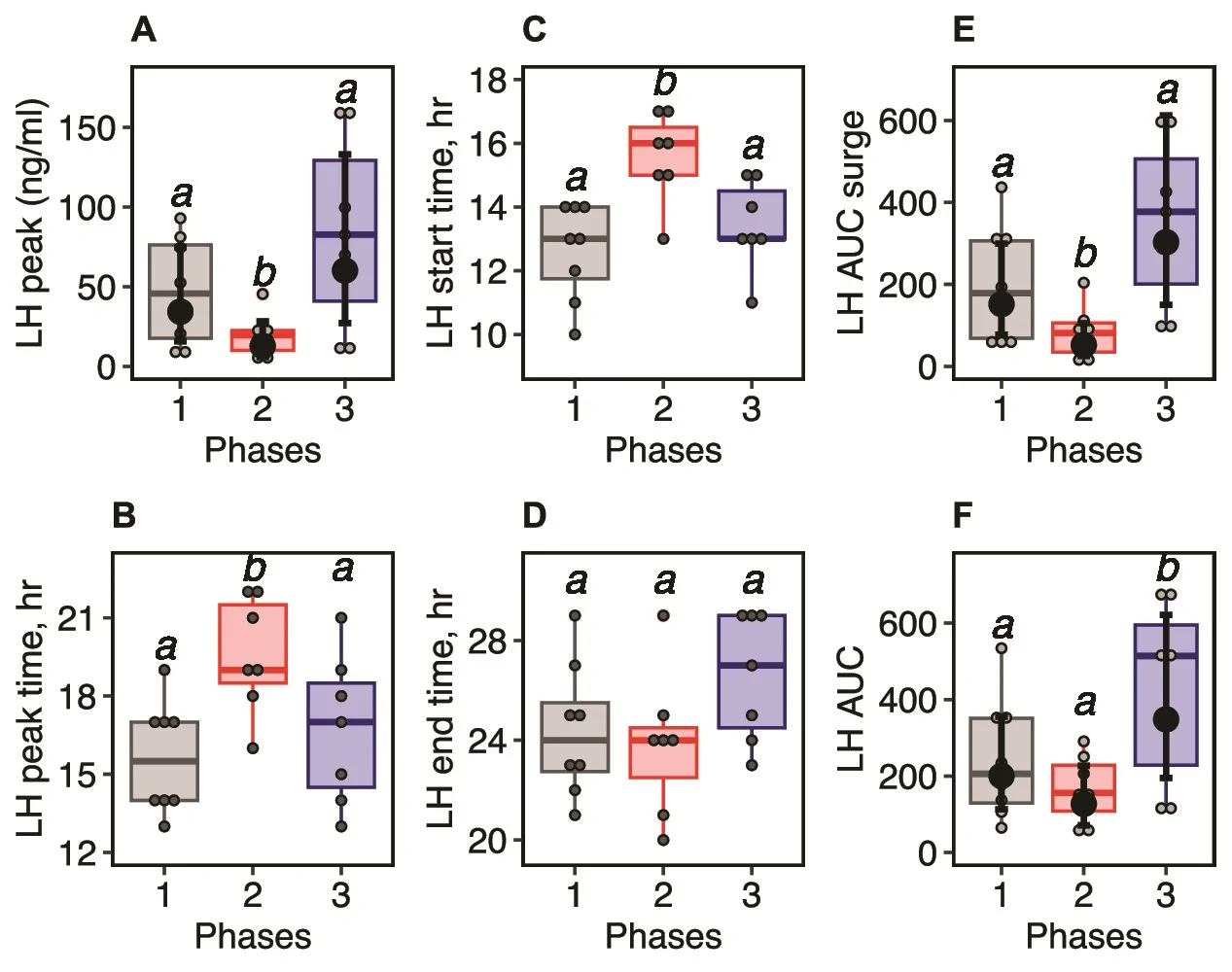
Obesity is associated with a delayed and blunted E2-induced LH surge. Serum LH concentrations were measured in the same ewes in phase 1 (normal weight), phase 2 (obese), and phase 3 (return to normal weight). Ewe LH values were averaged for each bleed time point and are represented as the means *+* SEM. These analyses are shown in (A) LH peak (ng/ml), (B) LH peak time (hr), (C) LH start time (hr), and (D) LH end time (hr). LH area under the curve during the surge is shown in (E), and total AUC is shown in (F). LH AUC surge analyses were conducted using a linear mixed-effects model, and letters indicate significant differences (*P* < 0.05). LH concentration and AUC were analyzed after natural log transformation. Values are back-transformed to the original scale. Box plots are presented as in Figure 3.

To examine the overall changes in the surge during all three phases, LH values were averaged across all ewes for each bleed time point during phases 1–3 (Figure 5). Subsequent analysis shows that the LH peak concentration is significantly lower (*P* < 0.01) and the timing of the LH peak is delayed (*P* < 0.001) in obese ewes as compared to values during phase 1 (Figure 5A and B). The LH surge start time is delayed in obese ewes compared to normal weight (*P* < 0.01), while there is no difference in surge end time (*P* = 0.99) (Figure 5C and D). Finally, the LH surge area under the curve (*P* = 0.02) is significantly different between phases 1 and 2 (Figure 5E). Total LH area under the curve was significantly greater in phase 3 compared to both phase 1 and phase 2, while phase 1 and phase 2 did not differ significantly (Figure 5F). At phase 3, the LH peak concentration (*P* = 0.15), timing of the LH peak (*P* = 0.52), LH surge start time (*P* = 0.45), and LH surge area under the curve (*P* = 0.12) are not statistically different from phase 1, indicating a restoration of LH surge parameters with weight loss (Figure 5A–E). As a control, E2 concentration was measured throughout the time course and reached a sufficient serum concentration to elicit an LH surge during all three phases of the study. Lastly, ewe serum cortisol was not significantly different across the three phases as measured at 0 and 27 h (data not shown).

## Discussion

Obesity has emerged as one of the leading health issues globally and in the United States, where it is estimated that >30% of the population is clinically obese [32]. One of the primary sequelae of obesity in reproductive-age women is impaired fertility, which is characterized by a relatively hypogonadotropic hypogonadal phenotype, exhibiting reduced secretion of LH, FSH, and progesterone during ovarian cycles and reduced pituitary sensitivity to GnRH [3, 5, 33]. Santoro and colleagues have named this phenotype reprometabolic syndrome, characterized by menstrual irregularities and a prolonged follicular phase [34]. Additionally, the reduced LH pulse amplitude and blunted response to GnRH in obese women implicate a functional impairment of the hypothalamic–pituitary–gonadal axis that is evident at the level of the pituitary gland. While these data suggest that a pituitary site of action may partially explain the inverse relationship between LH and body mass index, it is also clear that kisspeptin-expressing neurons integrate metabolic inputs associated with both under- and over-nutrition and thereby regulate GnRH output [35, 36]. Studying reprometabolic syndrome in women is particularly challenging due to the ethical and logistical barriers in accessing pituitary tissues and inducing obesity experimentally. Thus, despite the profound impacts of obesity on reproductive function, we still have a poor understanding of the underlying deficits at the level of either the hypothalamus or pituitary, and, currently, there are few options for alleviating the detrimental effects of obesity on women’s reproductive function, save for weight loss, as obesity impairs both natural conception and assisted reproduction [37–40]. An animal model of reprometabolic syndrome would provide a practical and ethical alternative for these investigations. Herein, we tested whether ewes can serve as a model of reprometabolic syndrome in women.

Ovulation is the central event in the female reproductive cycle and is dependent on a dramatic surge of LH driven by both pituitary and hypothalamic events. It is axiomatic that E2 derived from the preovulatory follicle serves as the most proximate regulator of the LH surge. The 16-day estrous cycle of the ewe [41] is like that of women, with the surge of LH resulting in ovulation. In a normal cycling female, E2 produced by cells of the preovulatory follicle is necessary and sufficient to signal to the hypothalamus and pituitary gland the readiness of the maturing oocyte and follicle for ovulation. The ewe has been an important model establishing that the preovulatory LH surge is the result of 2 key events: (1) greatly enhanced gonadotrope sensitivity to GnRH due to increased GnRHR expression, and (2) an increase in hypothalamic GnRH pulse frequency [22, 23, 42–46]. It is important to note that the E2-mediated increase in pituitary *GNRHR* expression is not only the initial event but also occurs directly at the pituitary and is not dependent on GnRH [47–49], creating an opportunity for both in vivo and in vitro study of pituitary responsiveness to endocrine input.

For over 40 years, both in vivo and in vitro ovine models have been seminal to our understanding of the sequence of events resulting in the LH surge [50]. Ovariectomized ewes will reliably produce an LH surge that starts approximately 12 h after administration of an E2 dose that mimics the peri-ovulatory period [22]. As discussed above, E2 acts directly at the pituitary to increase *GNRHR* expression, thereby enhancing pituitary sensitivity to GnRH. This is the earliest detectable event following E2 treatment and is evident within 4 h [23]. In contrast, the increase in GnRH pulse frequency in the portal circulation is temporally delayed [23]. At issue then is the identity of the lesions associated with obesity that result in a delayed and blunted LH surge observed in the present study. While the current study does not allow us to parse the relative impact of the obese phenotype at the hypothalamic and pituitary levels, it is likely that the positive feedback effects of E2 in the obese state may be compromised at both sites. As to the former, diet-induced obesity in rats has been associated with decreased Kiss1 levels and fewer immunoreactive Kiss1 neurons in the hypothalamus [51]. Although the impact of diet-induced obesity on the LH surge in sheep has not been previously reported, it is notable that central delivery of a KISS1R antagonist reduces LH surge amplitude [52] and, in at least one study, delayed the onset of an E2-induced LH surge [19], reflecting a dynamic that is similar to the phenotype of obese ewes in this study. As such, an impact on Kiss1 signaling to GnRH neurons may partially underlie the attenuated and delayed E2-induced LH surge we observed in phase 2 (obese). Similarly, however, a delayed and blunted LH surge is precisely what would be predicted if the ability of E2 to increase *GNRHR* expression was lost or substantially attenuated, thereby reducing pituitary sensitivity to GnRH. It is intriguing that reduced GnRH sensitivity and a reduction in LH pulse amplitude with no corresponding change in LH pulse frequency, as seen in women with reprometabolic syndrome, is also consistent with a pituitary rather than hypothalamic lesion [3]. Importantly, the sheep model will allow for both in vitro and in vivo approaches that are precluded in rodents and women to address the relative roles of hypothalamic and pituitary lesions associated with obesity. A prime example of this is the ability to sample portal blood, which allows a direct assessment of GnRH output from the hypothalamus.

The ovariectomized model clearly does not allow an assessment of the altered LH surge potential ovulatory deficits; however, anovulatory fertility [53] and reduced luteal progesterone production [3, 54] are associated with elevated Body Mass Index (BMI) in women. Equally important is the potential impact of a delayed LH surge in coordinated timing of final oocyte maturation, ovulation, and embryo quality [55, 56]. Finally, while possible, it is highly unlikely that the diminished LH peak in phase 2 animals reflects compromised E2 levels in the obese state. The dose of E2 administered in this study yields circulating levels of E2 that are 2 to 3 fold higher than those measured in late follicular phase ewes [23, 57]. Furthermore, in the same paradigm, lower doses of E2 may be associated with reduced peak LH response but the timing of the LH surge is not affected [58].

Regardless of the site of action, the other key question arising from these studies is the identity of the signal or signals that underlie the altered LH surge dynamics in the obese state. Certainly, adipose-derived signals such as leptin and adiponectin (or other adipokines) may play a role and have been implicated in reduced reproductive function in multiple rodent models [12, 59–63]. Similarly, obesity has been associated with a chronic inflammatory state [33, 64], raising the possibility that inflammatory signals could be at play. We would note that we did not see a generalized elevation in circulating levels of a panel of inflammatory cytokines (including TNF-α, IL-1β, CXCL9, and IL-17A), although IL-8 (CXCL8) and sFRP-3 (FRZB) were elevated in obese phase 2 sheep. However, we cannot preclude local elevations of these molecules in the hypothalamus or pituitary. Another distinct possibility, however, is that hyperinsulinemia evident in the obese state (Figure 2) may play a key role in the altered dynamics of the E2-induced LH surge. In support of this notion, work by Chosich et al. demonstrates that the combination of elevated serum insulin and free fatty acids suppresses LH and FSH, providing a potential mechanistic link between hypogonadotropic hypogonadism and obesity [65]. Further supporting this potential mechanism, a reprometabolic syndrome-like phenotype is recapitulated in normal-weight, eumenorrheic women by infusion of insulin and lipids [10]. Following weight loss via a low-calorie diet or bariatric surgery, ovulatory function improves and LH secretion partially recovers, although reproductive endocrine parameters often remain incompletely normalized [66]. This finding is consistent with our results, as the phase 3 LH surge amplitude for most ewes was significantly different from the LH surge amplitude during phase 2, although this trend was not consistent across all ewes. Thus, our results support that obese ewes are a representative model for further investigation of reprometabolic syndrome. In summary, our results suggest that ewes are a valid model to investigate the mechanisms occurring during obesity that alter the E2-induced LH surge associated with reprometabolic syndrome. In this model, obese ewes have an altered E2-induced LH surge that returns to normal following weight loss. This model represents features that are more similar to human reprometabolic syndrome than any obesity reproductive model observed in rodents. Further development of this model will allow investigation of whether the underlying mechanisms of disease are attributable to hypothalamic or pituitary sites of action and may facilitate the identification of potential therapeutic targets. As the global obesity epidemic increases, it is vital that we understand the causes of reproductive dysfunction, as it is a leading cause of female infertility. Our study establishes OVX ewes as a physiologically relevant model of obesity-induced changes in LH surge dynamics, providing a platform for future studies to investigate the molecular mechanisms by which obesity mediates these effects.

## Data Availability

Data supporting the findings of this study will be deposited in the Dryad Digital Repository upon acceptance. Analysis scripts are available upon request.
